# Microbiological Sensing Technologies: A Review

**DOI:** 10.3390/bioengineering5010020

**Published:** 2018-03-02

**Authors:** Firouz Abbasian, Ebrahim Ghafar-Zadeh, Sebastian Magierowski

**Affiliations:** Biologically Inspired Sensors and Actuators Laboratory, Department of EECS, Lassonde School of Engineering, York University, Toronto, ON M3J 1P3, Canada; fabbasian@cse.yorku.ca (F.A.); magiero@cse.yorku.ca (S.M.)

**Keywords:** biosensors, bacteria, microbiology, Lab on chip (LoC)

## Abstract

Microorganisms have a significant influence on human activities and health, and consequently, there is high demand to develop automated, sensitive, and rapid methods for their detection. These methods might be applicable for clinical, industrial, and environmental applications. Although different techniques have been suggested and employed for the detection of microorganisms, and the majority of these methods are not cost effective and suffer from low sensitivity and low specificity, especially in mixed samples. This paper presents a comprehensive review of microbiological techniques and associated challenges for bioengineering researchers with an engineering background. Also, this paper reports on recent technological advances and their future prospects for a variety of microbiological applications.

## 1. Introduction

Microorganisms are present everywhere and are involved in many clinical, industrial, and environmental phenomena. While the outbreak and richness of infectious diseases and food poisoning that are caused by microorganisms has declined through the years, the total numbers of these diseases is still highly prevalent [1,2]. Furthermore, the contamination of food/drug and cosmetic products with microorganisms or their derivatives remains a big challenge for industries operating in these markets. Present in all of these issues is the need for timely, ideally on-line, analyses of dangers, and compromised materials. As a result, there is a growing emphasis on the realization and application of real-time detection devices with high sensitivity and specificity for identification of microorganisms present in clinical/environmental/industrial samples [3,4]. This research approach has attracted the attention of engineering researchers to contribute in this field by proposing new solutions for current microbiological challenges. To date, many papers have reported the design and implementation of engineered microbiological protocols, such as microorganism biosensors [5,6,7,8,9]. With anticipation that such technologies will be dominated by miniaturized systems in the future, in addition to introducing the current microbiological practices to engineering researchers, we also review the most recent technological advances that are associated with this field of research.

**Figure 1:**
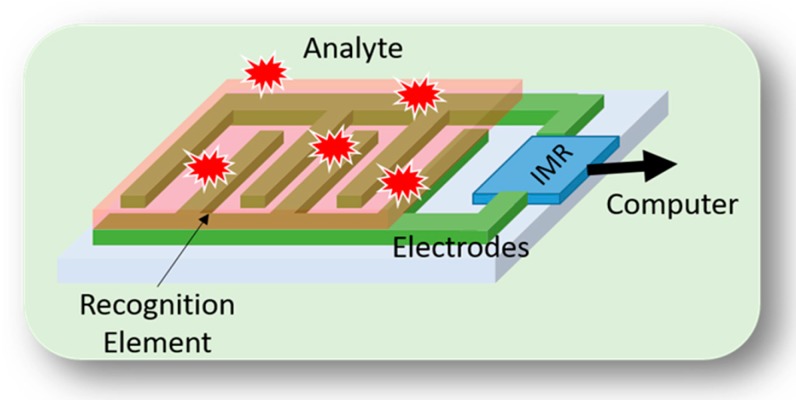
Illustration of a biosensor for the detection of microorganism including a recognition element which can be any specific agent, microelectrodes for impedance measurement, electronic impedance reader (IMR). The output of the IMR device is sent to computer.

Laboratory-on-a-chip (LoC) devices, or micro total analysis systems (µTAS), offer great advantages in terms of their material (e.g., reaction substrates) and time (i.e., rapid loading and analysis overhead) requirements. As a result, they are one of the most promising systems for the development of high throughput and automated biosensors [6,7]. LoC systems featuring a number of microscale reaction chambers and channels are used to prepare samples and to deliver analytes (e.g., bacteria, DNA, etc.) toward miniaturized embedded sensing sites [8]. The core part of a LoC system designed for microbial detection is a biosensor, which itself consists of a recognition element and a readout system [8]. The recognition elements, e.g., antibodies, bacteriophages, antimicrobial peptides, and bacteriocins, are used to convert the biological phenomenon to a physical or chemical variation (Figure 1) [10,11]. Indeed, any microbial features, or the presence of any specific factor in a special microorganism, including genomic elements, antigenic properties, electromechanical properties, metabolic activities, and/or photographic indexes are potentially useful for the detection of microorganisms in a sample [10]. The readout system or physicochemical transducers are used to sense, amplify, and measure the signals (mechanical, optical, electrochemical, and acoustic changes) obtained from the recognition element [12]. A readout system can be implemented using microelectromechanical system (MEMS) technology or microelectronic technology [13].

Among various competing microelectronic technologies for reading out the biosensors, complementary metal–oxide–semiconductor (CMOS) offers the capability of hosting complex analog and digital integrated circuits for signal processing and sensors for transduction on the same chip to track and analyze millions of changes in the nano/micro scale levels in a time and cost effective manner [13]. The potential for the mass industrial production of such CMOS devices coupled with high-performance data processing and machine-learning back-ends can make inexpensive and autonomous devices available to personal health care systems [13]. To date, many papers have reported the advantages of CMOS for a multitude of biological applications, including DNA sequencing and bacterial growth analysis [14,15,16]. However, the majority of these devices suffer from the inability to differentiate between living and dead microorganisms and fail to separate and identify the etiologic microorganisms in a mixed microbial sample [14,15,16]. Consequently, the small size of bacterial cells (typically 0.5–5 µm), and the multi-epitope characterization of the bacterial cell surface, restricts the application of routine biosensors for the detection of whole intake cells of microorganisms [13,17]. As a result of the aforementioned biosensing limits, the majority of researchers in this field have focused on the detection of pure bacterial cultures using optical and electrical readout systems [13,17].

LoC technologies can be split into culture-based and culture-free techniques. The culture-free systems rely on genomic and antigenic properties of microorganisms, and are limited to bench-top equipment that requires skilled technicians and are further hampered by the inability to differentiate between living and destroyed cells [18,19]. The principal for culture-based systems is, however, the measurement of changes in the electric and chemical properties of the microenvironment around the growing cells [20,21]. The main advantages of this system are direct detection of microorganisms without the complexity incumbent in techniques relying on fluorescent or genetic labels [20,21]. However, it must be taken into account that the selection of a method for the detection of microorganisms in a sample mostly depends on the nature of the microorganisms and their ability to respond to different chemical and physical treatments. It needs to be mentioned that there is the possibility of designing a detection device using a combination of these technologies to improve the quality and (maybe) the speed of microbial diagnosis.

The main focus of this paper is not placed on the recent advances of LoC, MEMS, or CMOS technologies for microbiological applications. Instead, the rest of this paper discusses the principles of the main microbiological methods. Among these methods, we will discuss the immobilization of bio-reporters on the surfaces for bio-sensing applications in Section 2. Also, the detection of intact microbial cells or a part of their cell contents, such as genomic contents, proteins, and fatty acids will be discussed in Section 3, Section 4 and Section 5. These sections will be followed up with a conclusion in Section 6. This section will also put forward the key challenges and critical future works in microbiological sensing technologies.

## 2. Immobilisation of Bio-Reporter on Functionalized Surfaces

Regardless of the technology used for the detection of microorganisms, the immobilisation of a biological particle on the surface of some measurement circuit is a real challenge. This step allows for the bio-reporter, which determines the specificity of a kit for a special target, to attach properly on the surface of the kit, and is therefore a crucial effort towards improved kit performance in terms of sensitivity, specificity and long-term stability [22]. Immobilization strategies differ mainly in the natures of the functionalized surface and the bio-reporter [22]. For different applications, the bio-reporter can be immobilized on different types of functionalized slide surfaces, including glass [23], silicon [24], PDMS (polydimethylsiloxane) [25], COC (cyclic olefin copolymer) [26], PS (polystyrene) [27], PMMA (polymethyl-methacrylate) [28], and metal films [29]. For the majority of these slides, the surface is first covered with a layer of a silanizing agent, such as siorganofunctional alkoxysilane molecules, which provide suitable functional residues for anchoring to functional groups (like -OH and -NH_2_) of proteins [30]. Park et al., for instance, used sulfosuccinimidyl 6-[3-(2-pyridyldithio)propionamido]hexanoate (sulfo-LC-SPDP) as a heterobifunctional cross-linker that was able to make a N-S band with an antibody (Ab) at one side and thiol reaction with an aluminium layer on the surface of the slide [31]. The selection of synthetic layers mostly depends on their properties, such as softness, optical transparency, chemical resistance, as well as their final cost and facilitation of fabrication [32,33]. In addition to the superiority of these options, the slides made of PMMA, PS, and COC show lower auto-fluorescence and contain intrinsic functional groups for the attachment of proteins [34,35].

In addition to the composition of the functionalized surfaces, the application of three-dimensional surfaces can increase the levels of protein coverage on the surfaces, and therefore, can improve the sensitivity of the device [36,37]. The dimensional surfaces are created by the formation of microbeads [38], hydrogels (such as polyacrylamide gel and polyethylene glycol) [39], porous membranes [40], micropits [41], and microposts [42]. While the same silanization technology is used to build up the functional groups on the 3-D silicon/glass surfaces, the polymer-based 3-D surfaces, including polymer monoliths, hydrogels, and agarose beads can be adjusted for immobilization using oxidative activation of functional groups, graft polymerization, and copolymerization of protein [22].

Along with the challenges of analyte attachment on the electrodes, the deposition of undesired particles on the surfaces, referred to as fouling phenomenon, is another serious situation needed to be addressed for the design of an efficient functionalized surface. This problem is normally resolved with the application of various physical and chemical methods. PDMS, for instance, is commonly used for fabrication of microfluidic devices used in biological and chemical analysis. However, this material shows very high hydrophobicity, which causes a biofouling property and low wettability [43]. The physical methods used to resolve this failure, such as interaction with electrostatic or hydrophobic interactions and surface activation by ultraviolet (UV) light, ozone, and oxygen plasma, are very temporary and susceptible to a return of the hydrophobicity property [44]. However, chemical modification, in which multiple reagents are used for the preparation of the slides, improves the immobilization due to the creation of covalent cross-bonds between the solid layer and the bio-reporters [44].

**Figure 2:**
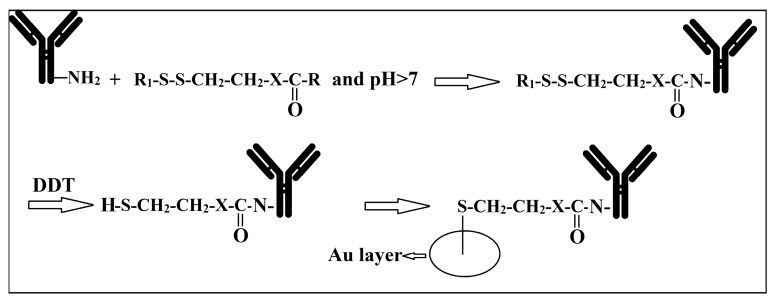
Antibody absorption on the surface of an electrode using thiolation cross-linkers. *R*1 and *R*2 are leaving groups; X is the residue remaining after antibody immobilization; DTT: dithiothreitol.

Several factors, including the properties of the bio-reporters, the nature of the functional surface, the chemical activities of additives, such as buffers and co-factors, and also, the sensitivity and specificity of kits affect the bio-reporter immobilisation strategy most suitable for a specific purpose [45]. A bio-reporter is immobilised on the surface using covalent bonding (with the sulfhydryl, amine or carboxylated groups of aminoacids), bio-affinity interactions (specific interactions, such as avidin-biotin, protein G-Antibody, DNA hybridization with its complementary DNA and also, aptamers), physiosorption (physical adsorption of bio-reports by low energy intermolecular interactions, such as hydrogen, Van Der Waals, hydrophilic, and electrostatic bonds), or a combination of all these three factors [45]. Da Silva et al. [46] immobilized biotinylated bacterial cells to slides coated with polymerized biotinylated functionalized pyrrole using avidin as the interacting molecules. However, immobilization of (especially) alive cells to a surface using avidin-biotin interaction can be prone to enzymatic/non-enzymatic disruption [47], and therefore, leads to release of the target cells from the surface (Figure 2). Aptamers, which are a group of synthetic/natural proteins/oligonucleotides with the ability to bind specifically to a target molecule, have been used for the design of biosensors since the 1980s [48] due to (a) high affinity to the target; (b) in vitro production; and, (c) their small size, that overall allows for the production of low cost and highly dense immobilized kits in comparison with other specific techniques [45]. Since the 1990s, many studies have been performed on the use of a single, or a cocktail, of these aptamers as effective means for design of microbial detection sensors capable of making strong bonds to different components of microbial cells, such as cell wall, cell membrane proteins, flagella, extracellular enzymes, or genomic contents (RNA/DNA) [49,50,51,52,53,54]. Application of a cocktail of these molecules improves the target attachment to the biosensors [49]. However, in case of either a single aptamer or a cocktail of these aptamer, these factor should not affect the conformational structure and functionality of the bio-reporters [45]. Application of heterobifunctional cross-linkers, which work as bridges to create large spacing between the cells and the surface, facilitates cell-Ab interactions on the electrode [31].

## 3. Detection of Intact Cells

Phenotypic analysis refers to the preliminary step of the identification of microorganism’s observable traits. Conventional methods distinguish cellular subjects based on morphological features, such as: size, shape, motility and number of flagella, sporulation, shape and position of spores in the cytoplasm, encapsulation, inclusion bodies, staining features, and surface and ultrastructural characteristics. Cellular colonies are also distinguished based on: form, size, elevation, margin, opacity, consistency, and pigmentation [55]. Microscopic studies for detection of intact microbial cells, which primarily need staining before observation, are time-consuming and less specific [56,57], and it is not possible to use this technique in LoCs unless in combination with a reporter system, such as fluorescence.

**Figure 3:**
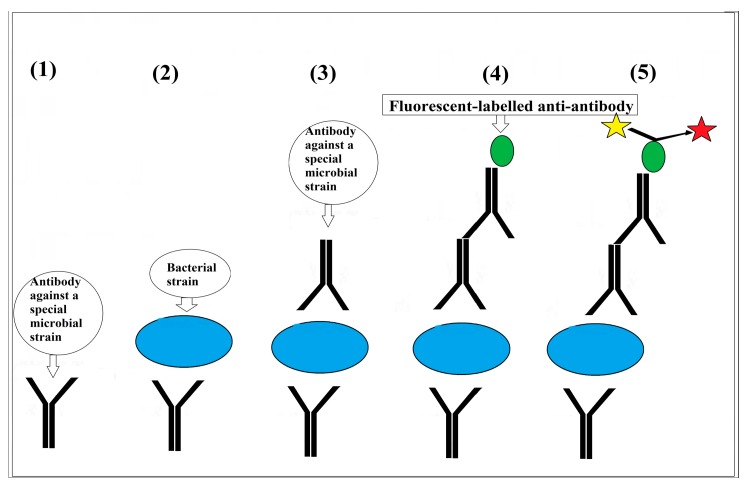
Sandwich technique: in this method, a specific antibody may be used to fix the particle on the surface of a chip. Attachment of another specific antibody on the free site of the particle can be recognised by the addition of a fluorescent-labelled anti-antibody (such as anti-IGM and Anti-IgG).

The biosensors used for the detection of intact bacterial cells commonly employ different optical, electrical and chemical detector techniques. The optical technologies mostly rely on the traditional sandwich technique used for immunoassay analysis, in which the bacterial cells are specifically captured by the antibodies that are immobilized on the kit, and the captured cells are recognized by a second specific antibody conjugated to a fluorescent/luminescent dye [9,58,59] (Figure 3). Myong song and his colleagues for instance, used laser induced fluorescence (LIF) for recognition of the antibody-bacterial cell (*Bacillus globigii*) reactions in a CMOS system [60].

**Figure 4:**
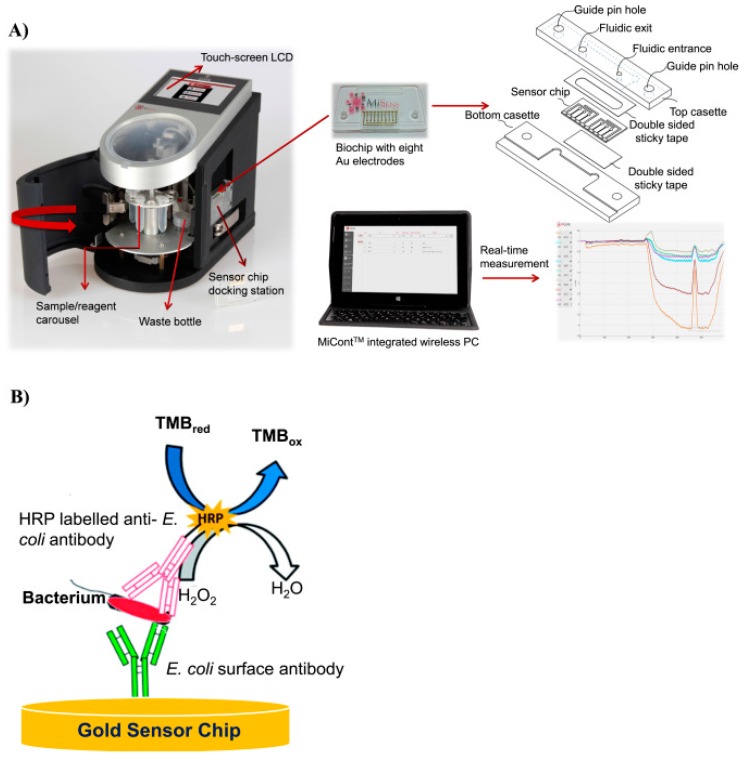
The design of a microfluidic device based on a horseradish Peroxidase (HRP)-conjugated biosensor for detection of *E. coli.* Here, the target bacterial cells bind to their specific antibodies on the gold sensor chip. The bacterial cells bonded to the biosensors are recognized by the addition of another strain-specific horseradish Peroxidase (HRP)-conjugated antibody. A washing step between each sample or antibody addition will remove all unbounded cells or (HRP)-conjugated antibody, and therefore, any enzymatic reaction in the media occurs as a result of the presence of the antibodies bonded to the cell [62]. The enzymatic reaction of H_2_O_2_ to H_2_O reduction is associated with oxidation of TMB (3,3′,5,5′-Tetramethylbenzidine), which can be detected by very sensitive gold electrode (This Figure is used from Elsevier with permission).

It is possible to apply Fibre Optic Biosensors (FOBs) in the sandwich structure, in which the antibodies are immobilized on an optical fiber and cells consequently attached to them. The binding of a secondary labeling factor to this overall complex will change the optical signal passing through the fiber [61]. Altintas and his colleagues, for instance, were recently able to use a combination of a microfluidic device and a horseradish Peroxidase (HRP)-conjugated biosensor to design an automated labour free device for the detection of *E. coli.* The electron required for the reduction of H_2_O_2_ into H_2_O by peroxidase is supplied by TMB (3,3′,5,5′-Tetramethylbenzidine), and all the electrical changes can be detected by a sensitive gold electrode (Figure 4) [62]. Yao et al. used a bacteriophage as the specific biosensor for the detection of *E. coli* in a CMOS based integrated sensor system [63]. Nikkhoo and her colleagues were able to improve the sensitivity of the bacteriophage receptor with the employment of integrated ion-sensitive field-effect transistors (ISFETs) implemented in conventional 0.18 μm CMOS with additional post-processes, most notably a PVC-based potassium-sensitive membrane atop the chip [64]. Mejri et al. indicated that the application of a bacteriophage for the detection of a bacterial species is more specific and accurate than the use of antibodies since a phage is able to generate successive dual signals of opposite trends over time [65]. Specifically, the initial increases in the impedance due to the attachment of the bacterial cells to the phages is followed by a sudden decrease in this value due to bacterial cell lysis (as a result of lytic activity of phages) [65].

**Figure 5:**
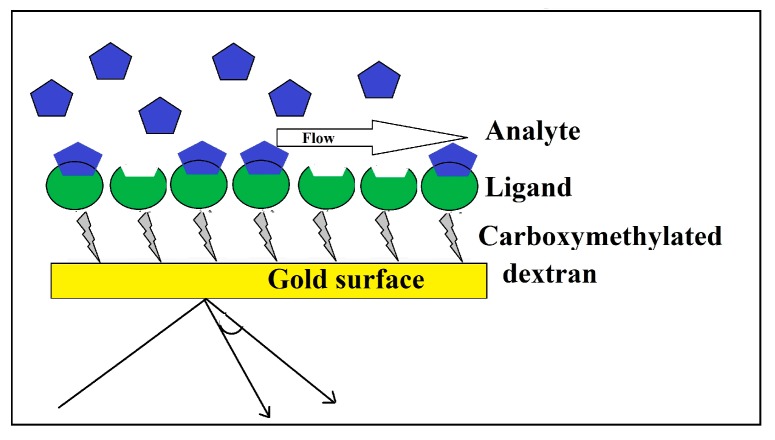
Surface Plasmon Resonance (SPR); the analyte binds to its specific ligand on the surface of a glass prism. This interaction is detected by passing a plane-polarized light through this film to a transducer surface which produces electrical pulses.

The development of different high affinity molecules, such as antibodies, phage, and other natural/synthetic factors for the detection of specific moieties, such as antigenic epitopes, receptors and specific hydrocarbon, respectively, on the surface of bacteria have been improving optical based devices for the detection of microorganisms [66]. Furthermore, scientists have been focusing on a way to resolve the limitation of the sample preparation step using the application of so-called label free optical methods, such as Surface Plasmon Resonance (SPR) (Figure 5) [67]. In SPR systems, plane-polarized light passes through a glass prism to reach a transducer surface, which, in response, produces electrical pulses. Attachment of specific receptors (such as an antibody, lectin, and bacteriophage) to this surface makes this transducer specific to a special type of analyte; any transducer-analyte binding will be associated with changes in the electrical pulse [67]. Bouguelia et al., for instance, were able to use this system for the label-free and real-time monitoring of single cell multiplying bacteria on a biochip [68]. Yoon et al. employed radiofrequency (RF)/microwave microstrip bandpass filter circuits in which the bacteria present in water samples were trapped atop the planar filter [69]. The bacterial cells so trapped change the relative permittivity of the insulating material, thus altering the filter’s frequency response. However, this system is only able to detect the presence of microorganisms and is not able to differentiate between different types of microorganisms. Therefore, such systems are not yet of immediate benefit in routine microbiology laboratories for detection of various microorganisms. While immobilization of an array of specific antibodies on such chips and analysis of the outgoing data from this system can be applied for the specific detection of single or multiple known microorganisms even in a combined contamination or complex environmental or clinical sample, this chip is applicable for detection of only the bacteria that are specific for those antibodies.

Application of mechanical biosensors, such as cantilever technology [9,70] and quartz crystal microbalance (QCM), are new strategies to improve the sensitivity and final costs of LoC devices [71]. In combination with a specific receptor and a cantilever sensor with oscillation at a certain resonance frequency, micro-cantilever-based devices measure changes in the resonance frequency caused by the binding of specific cells to the receptors [72]. With a similar strategy, QCM detects the micro-changes in the total mass of the receptors after bonding of a cell to its specific receptor, itself attached to a piezoelectric biosensor [73]. Miniaturization of such equipment will reduce the massive size of the device and, therefore, will make these devices affordable for LoC purposes [74]. However, it must be mentioned that all such devices designed to-date are able to detect only a single bacterial strain, and therefore, application of these devices needs prior information about cell properties, especially their antigenic features.

**Figure 6:**
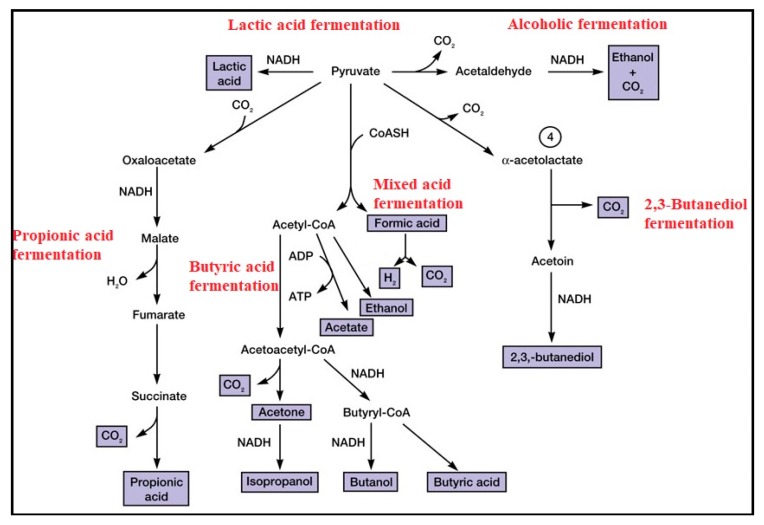
Schematic description of microbial fermentation.

**Figure 7:**
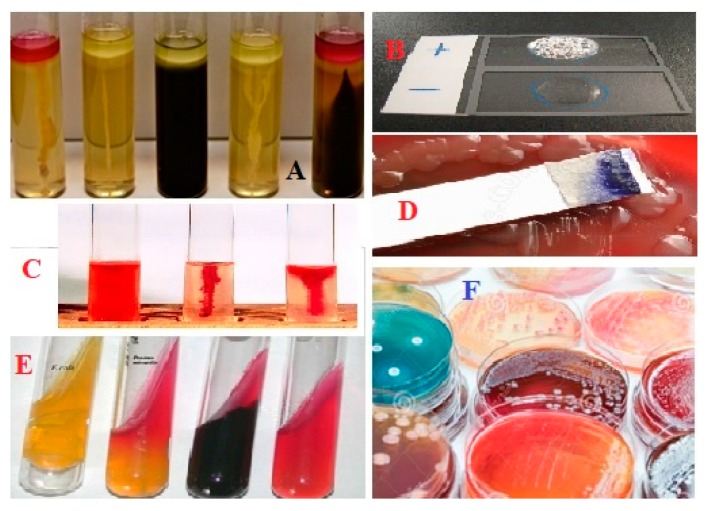
Metabolic activities of microorganism are the most important factor for the detection of microorganisms in traditional bacteriology; **A** = SIM (Sulfide-Indole-Motility) media; **B** = Catalase; **C** = SIM media with different bacterial inoculation; **D** = Oxidase test; **E** = TSI (Triple Sugar Iron Agar); **F =** different plate agars.

In addition to their physical features, microorganisms are detectable based on their metabolic and biochemical reactions as well as their physiological characteristics [56,57]. To use these features for identification, microorganisms are grown in microbial media and are subject to special tests based on the enzymatic activities, microbial growth requirements, microbial resistance to special chemicals or physical factors and microbial fatty acid composition [56,57]. Conventionally, biochemical tests are used for the differentiation of bacteria based on their genetic abilities to metabolize and convert special groups of substrates [56,57]. The cultivable bacteria are grown in a series of different media, each containing a specific substrate. Thus, their ability to use different substrate(s) (carbon, nitrogen, and sulphur), their ability to produce any sort of fermentative products, their sensitivity to metabolic inhibitors (such O_2_, H_2_O_2_, temperature, pH and osmotic pressure) and antibiotics, and their response to the production of degrading enzymes are investigated based on observations of changes in the appearance, texture, or color of the media (Figure 6) [56,57]. The final decision for detection of a cultivable bacterium is made based on an analysis of the microbial response to a combination of different metabolic and growth abilities [57]. Indeed, microbial responses to a complex of detection criteria are exclusive to a specific strain and can be used as a signature for that strain. The culture-based analysis for bacterial detection is reported based on observations of the physical/chemical changes in the media (such as changes in the color, turbidity, the production of gas, and so on) after an overnight incubation for the growth of bacteria, which makes these tests too time consuming and occasionally unreliable (Figure 7) [75].

**Figure 8:**
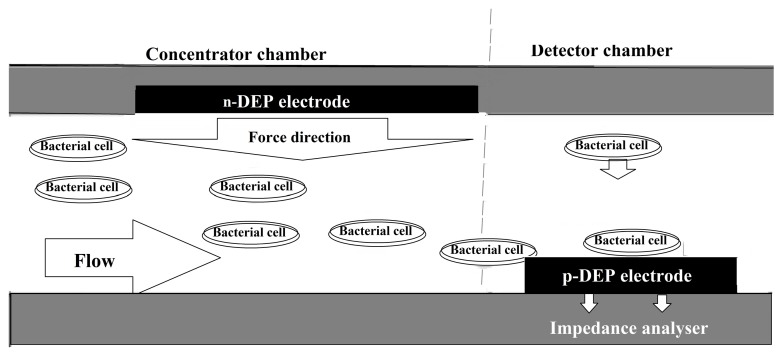
A microfluidic system to direct and concentrate bacterial cells on an electrode based on both flow and dielectrophoresis. The cells collected at the positive side of electrophoresis screen are detected by an impedance analyzer.

The employment of LoC devices incorporating electromechanical biosensors for data analysis provides the possibility of designing very sensitive, cost effective and fast approaches to sense any slight changes in the microbial growing media, which are related to microbial activities [6,7,76]. The BacT/Alert system (Figure 8), for instance, is a CO_2_ production-based instrument that is used for detection of blood infections caused by different types of bacteria [75]. In this system, the production of CO_2_ in aquatic media produce H_2_CO_3_- and H^+^, and the interaction of free hydrogen with a pH sensor leads to the generation of a voltage signal. These systems however, are only able to detect the presence of microorganisms in a given sample, while the type and diversity of microorganisms have to be detected by further microbial analysis. 

To cope with these problems, electrochemical biosensors have recently been included in the design of new versions of commercial LoCs [77,78]. The advantages of these systems are their capacity to implement label-free, cost-effective, real-time, miniaturized and highly sensitive devices [77,78]. The strategy behind the electrochemical sensors is to measure the electrical changes including voltage (potentiometery) or current (amperometry) or impedance (impedometry) [76,77,78]. Impedimetric biosensors rely on the electrophysiological nature of microbial cells and therefore work based on changes in the electron transfer resistance and capacitance following cell attachment to the electrodes or cell lysis [79,80]. Unlike the highly conductivity nature (up to 1 S/m) of the cytoplasm, the bacterial cell membrane is highly insulated (with an average conductivity of 10^−7^ S/m) [81]. Therefore, while cell attachment reduces the impedance of a particle, cell lysis can cause increases in this index [82,83]. This is the reason why impedimetric studies of the whole bacterial cell in deionized (DI) water lead to better responses in comparison to those in PBS (phosphate buffer solution) [79,80] which is more conductive and hence blurs impedimetric contributions from the cell material under test. Impedimetric biosensors can be made specific for certain types of microbial cells following association of the electrodes to specific receptors, such as antibodies and bacteriophages [79,80]. The impedimetric value of these interactions is categorized as either Faradaic, when the interactions are detected by a mediator such as Ferricyanide and Hexa-ammineruthenium III/II, or non-Faradaic, in the absence of a mediator. The results of microbial detection based on impedimetric analysis depend on the size and shape of the analyte, the absorbance affinity of the chosen bio-receptors, the way of presenting data (such as absolute/imaginary/real impedance and raw/percent R_ct_ changes) as well as the materials used for electrodes and the base layer [84,85]. This technology has been used for the detection of microorganisms since the 1970s, and there are many reports of using this factor for microbial analysis in food products, clinical specimens and environmental samples [84,85]. While the application of impedance factor as the only detective factor is not specific for detection of microbial species, the integration of polyclonal antibody and electrochemical impedance spectroscopy (EIS) converts this system very specific for certain microbe(s) [86]. In the experience performed by Maalouf et al., the antibody-conjugated EIS was more sensitive than SRP for the detection of *E. coli* (10 cfu/mL versus 10^7^ cfu/mL, respectively) [86].

**Figure 9:**
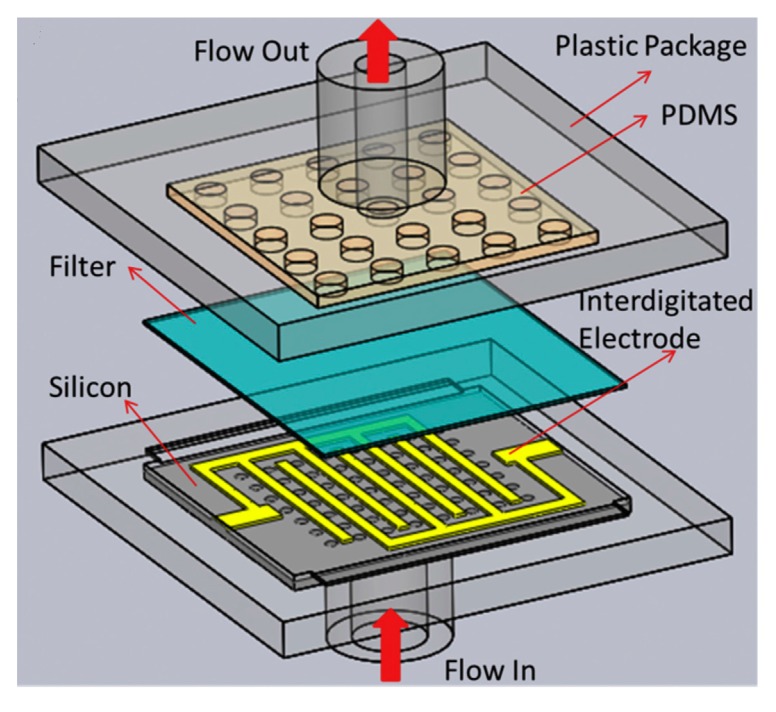
Cross-sectional view of the integrated the electrochemical impedance spectroscopy for detection of bacteria; in this system, bacterial suspension are flown into a nanopore filter above the electrodes where the cells trapped in the filter and the rest of the suspension is flown out [90] (This Figure is used from Elsevier with permission).

The impedance technology for detection of microorganisms can be boosted by integration of dielectrophoresis (DEP) to the chip, which enables the system to concentrate the microbial cells from a diluted microbial sample [87,88]. In this system, a microfluidic device is designed in such a way that microbial cells are repelled by both a liquid flow force and a negative DEP to be deposited on a positive DEP situated at the downstream of the device. The microbial cells that are concentrated on the positive DEP are detected by an impedance analyzer (Figure 8) [88]. This system can be specific for the detection of a certain microorganisms in a microbial mixture if the positive DEP is covered by specific antibodies [89]. Suehero et al., for instance, applied this strategy to direct a mixture of microorganisms to the antibody-conjugated microelectrodes. A rinse step following the binding of the bacterial species specific to the antibodies removes all of the non-target bacteria, and therefore, this system was specific for detection of certain bacterial strains in a mixture of microorganism [89]. In another strategy to improve the sensitivity of impedance-based detectors, Jiang and his colleagues designed a microfluidic device in which the microbial solution is passed through a filter where the microbial cells trapped in the filter are detected by an integrated electrode located below the filter (Figure 9) [90]. The sensitivity of this device was claimed to 10 bacterial cells per millilitre.

Amperometric-based technologies measure the changes in the current of a microbial media, which are directly proportional to the concentration of reactants or microbial cells [77]. The amperometric index of microbial media changes as a result of microbial metabolism which leads to the release or absorption of ionic metabolites or elements [77]. Potentiometric devices function based on recognition of analytes by specific ion electrodes, which is trackable based on changes in the potential of the microbial growth media [91]. Potentiometric technologies have been used for whole cell detection of a few bacterial cell types, such as sulfate-reducing bacteria [SRB] [92] and *Staphylococcus aureus* [93] by potential stripping analysis (PSA) and electromotive force (EMF) technologies, respectively.

**Figure 10:**
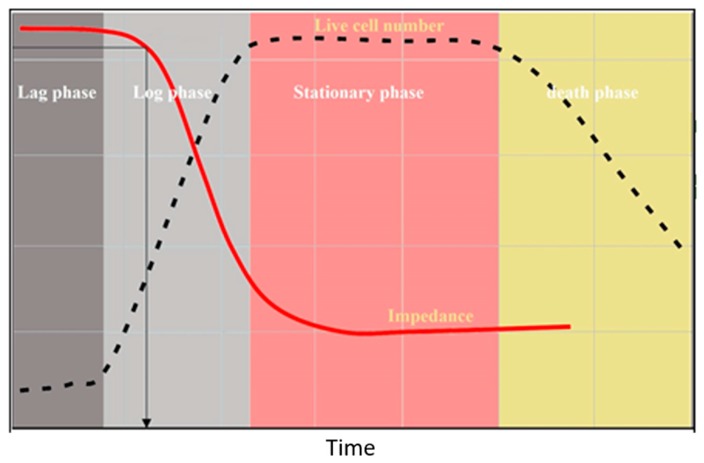
Bacterial growth curve. The lag phase after inoculation of a media with bacteria is followed by a logarithmic growth of the bacteria (dashed line). The shortage of nutrients and the production of toxic compounds in the media lead to a decline in the microbial growth during the stationary phase and to death at the last steps. The number of cells in this media is inversely correlated with the media impedance (solid line).

Commercially available bactometer devices use a combination of conductance, capacitance, and impedance to perform several microbial analyses per hour [94], and are able to perform microbial analysis on different types of microorganisms, including bacteria, fungi, viruses, algae and protozoa. The changes in the output electrical signals throughout microbial growth depend on the nature of media, microbial primary inoculation, microbial doubling rate, and the presence of optimal physicochemical condition, including pH, temperature [85]. The threshold time for each microorganism is the time that the electrical index curve starts to accelerate [95] (Figure 10), and starts in a concentration of 10^7^ bacteria (2–11) and 10^4^–10^5^ yeast (2–10) per millimeter. Since the electrical changes of a microbial environment are influenced by the metabolic activity of microorganisms, the nature of microbial media is a significant factor for more sensitive detection of microorganisms [95].

Overall, real-time analysis of microbial growth and the possibility of miniaturization of these devices are the most outstanding advantages of the application of electrochemical biosensors for point of care studies [85]. Furthermore, There is the possibility of measurement of the number of cells in a given sample based on measurement of the output electricity [85]. Besides, based on changes in the electrical indices of a given microorganism in the presence of a growth inhibitor, this technology is able to determine the susceptibility of bacteria to growth inhibitors such as antibiotics which can be used for antibiogram tests (a routine test performed by microbiologists in medical laboratories to determine the level of microbial sensitivity to an antibiotic) [96]. These technologies are especially useful to accelerate the detection of slow metabolic activities, as can be seen in slow-growing microorganism, such as *Mycobacterium* sp. [97,98] or in some anaerobic microbial activities, such as microbial desulfurization [92]. However, any innovative devices for bacterial detection based on the electrochemical activity must resolve the interference caused by other bacterial strains present in bacterial samples, especially in environmental samples or many clinical samples such as stool and sputum. 

On the other hand, while application of the sandwich method based on specific reagents (such as antibodies, bacteriophages, receptors, enzymes and so on) in conjunction with an electrochemical detection factor makes the detection devices very specific for a particular type of microorganism [99,100], these approaches fail to detect the presence of multiple microorganisms in a sample. Furthermore, in the case of usage of multiple specific antibodies for multiple microbial detections, these (usually gold-made-) chips are disposable and therefore, are too expensive for the design of point of care LoCs [99,100]. However, the design and application of newly emerged technologies related to the structure of the base matrix and electrodes, such as the nanoporous membrane-based impedimetric immunosensor [101], reduced graphene oxide paper [102], platinum wires [103], nickel foam [104], can improve the sensitivity and specificity of these devices. Immobilization of antibodies on the surface of the nanoporous membrane using an adherent, such as hyaluronic acid, makes a very specific binding surface upon which that attachment of bacteria changes the ion permeability through the membrane, with its effect being traceable by electrical sensors [105].

**Figure 11:**
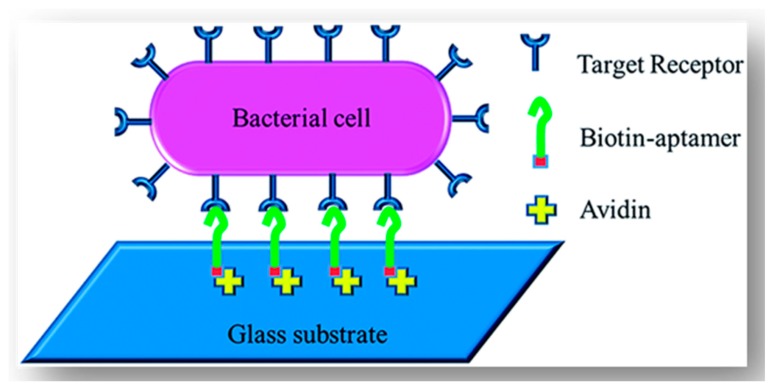
A simple schematic description of the employment of aptamers for the detection of microorganisms. In this condition, the complex of Biotin-aptamer works as a specific intermediate between the microbial strain, which is free in the media) and Avidin, which is stabilized on the surface. The aptamer complex is usually bounded to a recognition marker, such as a fluorescent).

The integration of aptamers (small peptide or oligonucleotide molecules with the ability to bind specifically to a target molecule) with a detection system is another strategy to achieve more specific detection of microorganisms even in a mixture of microorganisms [93]. Application of different detection factors in such systems can provide a detection chip for multiple pathogenic microorganisms. Wu et al., for instance, used multi-color (luminescent) up-conversion nanoparticles (UCNPs) in combination with specific aptamers for each bacteria strain (overall three strains) to design a sensitive chip for simultaneous detection of three bacteria in a complex (food) environment (Figure 11) [106]. In this case, however, an increase in the numbers of strains intensifies the luminescence interferences, and therefore, decreases the sensitivity and specificity of the kit. Furthermore, the number of strains detectable in this system is limited to the number of known aptamers specific for this microorganism.

## 4. Genomic Based Analysis

Microbial genomic studies are currently the most popular molecular identification and classification method of microorganisms in different types of samples based on the selection of one or more specific molecular target(s), such as DNA, RNA and protein, without the requirement to grow in their culture media [107]. Genetic microbial detection assays can be classified into genome GC% (Guanine + Cytosine) contents, pattern amplification (polymerase chain reaction; PCR, and loop mediated isothermal amplification; LAMP), DNA/RNA hybridization, genome polymorphism (such as RFLP: restriction fragment length polymorphism and AFLP: amplified fragment length polymorphisms) and gene sequencing [108,109,110]. In addition to the sample preparation and genome extraction, the majority of these methods need a nucleic acid amplification and an amplicon analysis step [110]. The sample preparation and DNA extraction steps are very challenging steps due to the removal of inhibitors from genomic samples and accurate measurement of the initial genomic contents per specific amounts of sample, which is used in quantitative methods [110]. These samples are always passed through an enrichment step, which can be performed by overnight incubation of bacteria in a specific sample (only limited to cultivable bacteria), centrifugation, and immunomagnetic separation [108,109,110]. These two latest samples can be applied for the removal of sample matrix from the microorganisms, which indeed is useful for removal of inhibitors from the genome [108,109,110]. A combination of conventional alkaline lysis protocol with DNA binding to silica or its derivatives has provided the possibility to design a cost effective automated microfluidic device for DNA isolation [111]. Bavykin et al., for instance, have developed a technique in which cell lysis is carried out in the presence of a high chaotropic agent, such as guanidine thiocyanate (GuSCN), followed by cell lysate injection through a universal syringe-operated silica micro-column to capture the RNA/DNA contents [112]. 

### 4.1. Guanine-Cytosine Contents

The genome GC% contents are the percentage of the sum of guanine and cytosine in DNA or RNA of a cell. While the GC% is markedly variable in different types of microorganisms, 17–75% [113], it cannot be used as the only criteria to distinguish microorganisms. Indeed, GC% is a useful factor to detect microorganisms along with a combination of other genetic/phenotype characterizations.

### 4.2. Sequence Amplification Techniques

**Figure 12:**
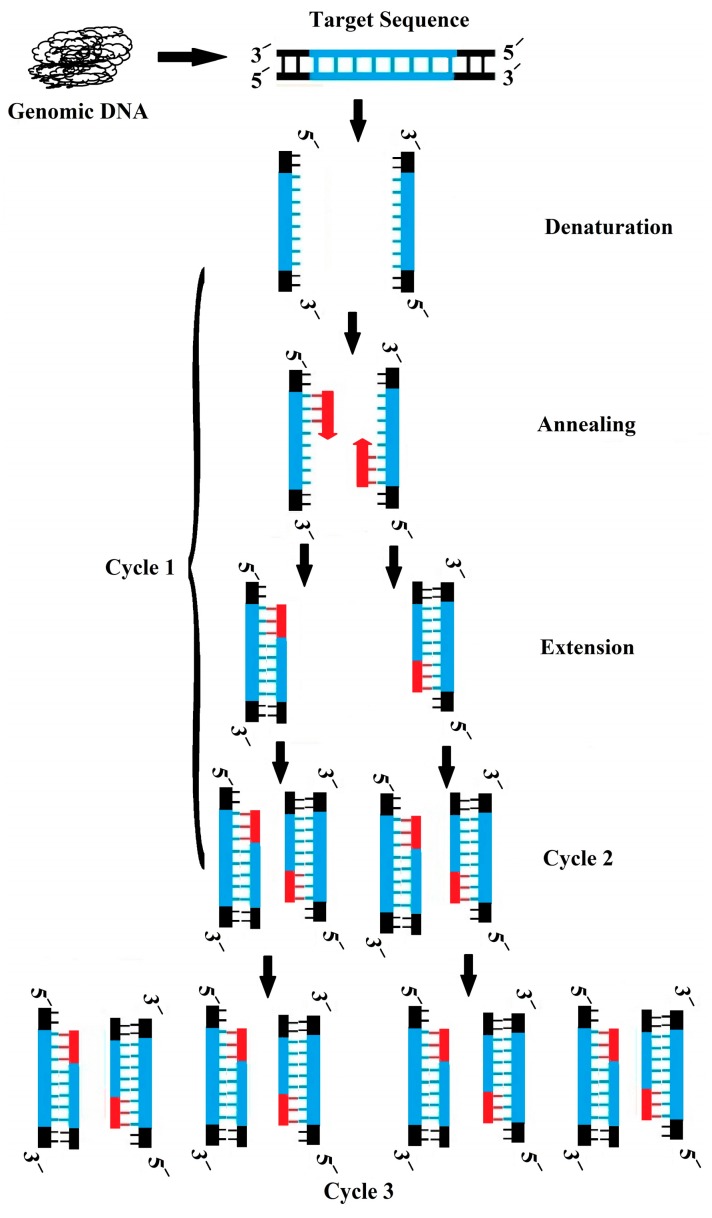
The principle of polymerase chain reaction (PCR); through denaturation period, increase in the temperature of the media (more than 90 °C) lead to separation of the DNA duplex in the form of single strands. Reduction of temperature to around 50 °C (depending to the primer) in the annealing step provides the condition for the single strand nucleic acids, including the primers, to re-hybrid in the form of double strand DNA. Thereafter, though extension step, an amplifier enzyme is able to use the 3′ end of the primer to extend the primer based on the complementary template DNA. These steps are usually repeated more than 30 times (depending on the concentration of the template DNA) to increase the numbers of the fragment sufficient for visibility on a gel. Since the primers are specifically designed for a special fragment of the genome, the amplification is specifically performed on that area of the genome.

PCR is a technique in which one or more specific fragments of the genome are amplified using specific primers, which are finally detected based on the size of the fragment in conventional PCR, polymerase chain reaction, or fluorescent reporters in real-time PCR [114]. In conventional PCR (Figure 12), the amplified fragment moves under a special electrical charge that separates the fragments based on their size and thus yields one or more identifiable band(s) onto the gel; the correct band among all of the other unspecific bands is determined in comparison with the size marker, which is run onto the gel at the same time [110].

**Figure 13:**
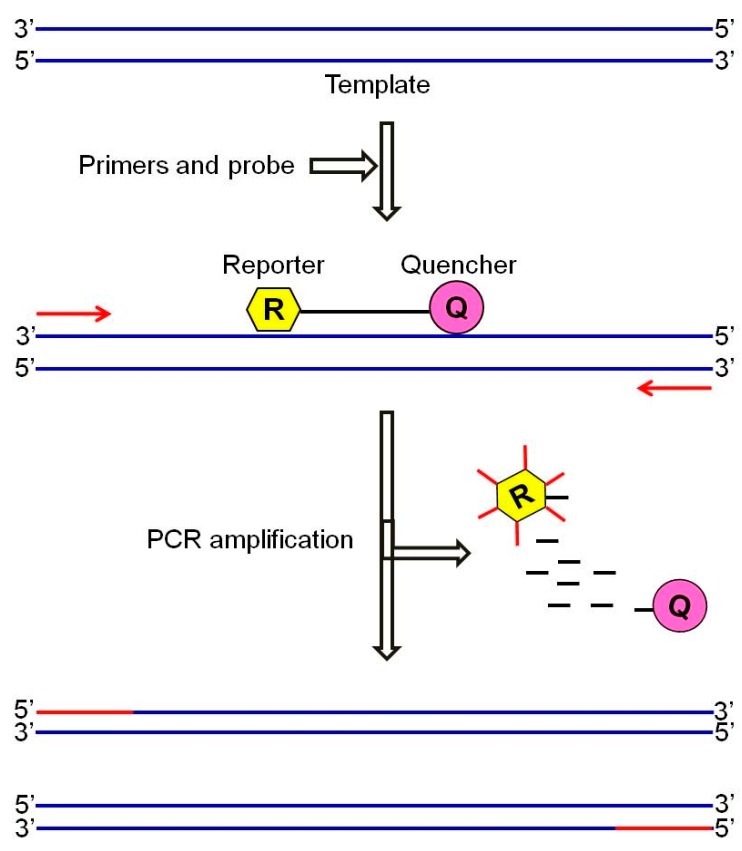
The real-time PCR; in this system, a specific probe labelled to two types of fluorophores, referred to as R for reporter (donor) and Q for quencher (or receptor), sits in the interval of two primers. When the distance between two fluorophores is between 1 and 10 nm, any the excited energy of the R is donated to Q in the form of Fluorescence Resonance Energy Transfer (FRET). Through amplification of the DNA, the R is cut by the amplifier enzyme, which lead to the separation of R and D fluorophores and emission of fluorescence. The level of emission is linearly proportional to the number of probe and genome.

Because of the ocular observation-based analysis, this technique cannot be fit in the LoC technologies and automation unless the primers are linked to a (fluorescent/radioactive/enzyme) label. Real-time PCR or quantitative PCR (q-PCR) (Figure 13) is a more sophisticated technology to characterize the amplifying reaction in time and to collect the data throughout the amplification process [115].

**Figure 14:**
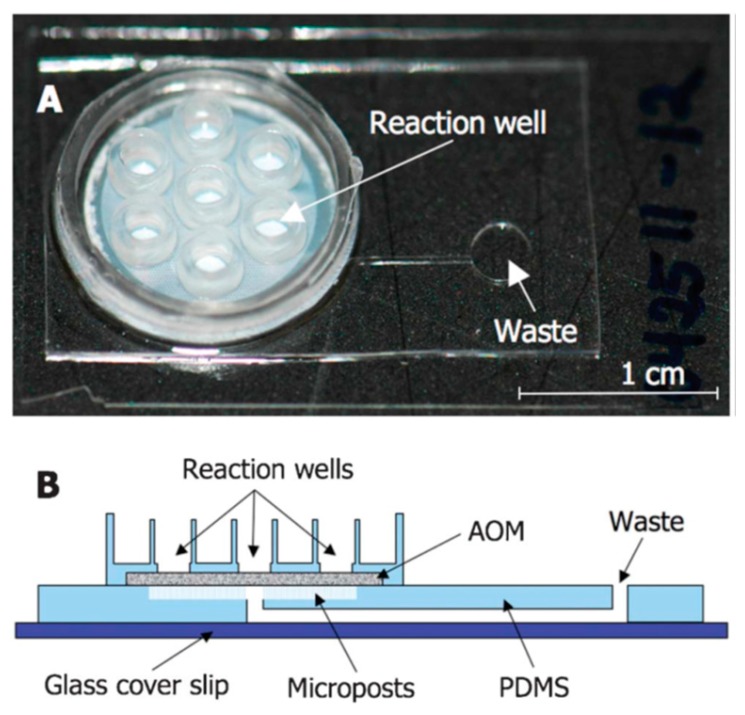
The microbial cell lysate is passed through an AOM filter to separate DNA on the surface, which finally undergoes a RT-PCR reaction [117]. (This Figure is used from Royal Society of Chemistry with permission).

In this system, target specific fluorescent–labelled probes are bound to the target al.ong with the primers, and any single target replication through each single PCR reaction leads to the release of the fluorescent reporter, which is associated with illumination of a fluorescent light [115]. Since each fluorescence is parallel to one gene amplification, the numbers of fluorescent pulses is a reliable factor to determine the copy numbers of genes based on comparison with the reference samples, which contain a defined number of the gene of interest and are run along with the samples of interests [115]. Employment of probes linked to different types of fluorescent reporters enables researchers to use qPCR for simultaneous detection of multiple microorganisms in a sample [116]. The ability to monitor the illumination emitted from the reporters makes qPCR suitable for the design of automated detection systems. Oblath et al., introduced a microfluidic chip with the ability to perform both DNA extraction and real-time PCR (Figure 14) [117]. The microbial samples were lysed by a heat treatment before addition to micro-wells, and were filtered by an AOM nanofilter to concentrate the DNA samples on the filters. The genomic materials that remained on the surface of the filters underwent a RT-PCR reaction. However, it needs to be mentioned that the huge amount of cell debris through the DNA/RNA extraction can affect the quality of RT-PCR performance, therefore a suitable genome extraction microchip should be designed in a way to separate this cell debris. Furthermore, it must be mentioned that the PCR procedures are very expensive and time-consuming, and therefore, is not presently amenable with the spirit of real-time microfluidic devices which need to be inexpensive [118].

**Figure 15:**
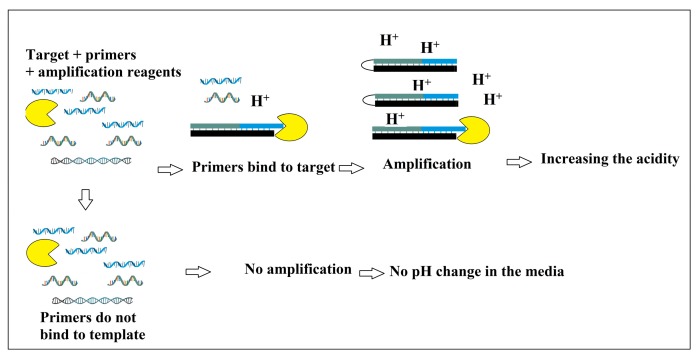
Loop mediated isothermal amplification (LAMP) method (description in the text).

**Figure 16:**
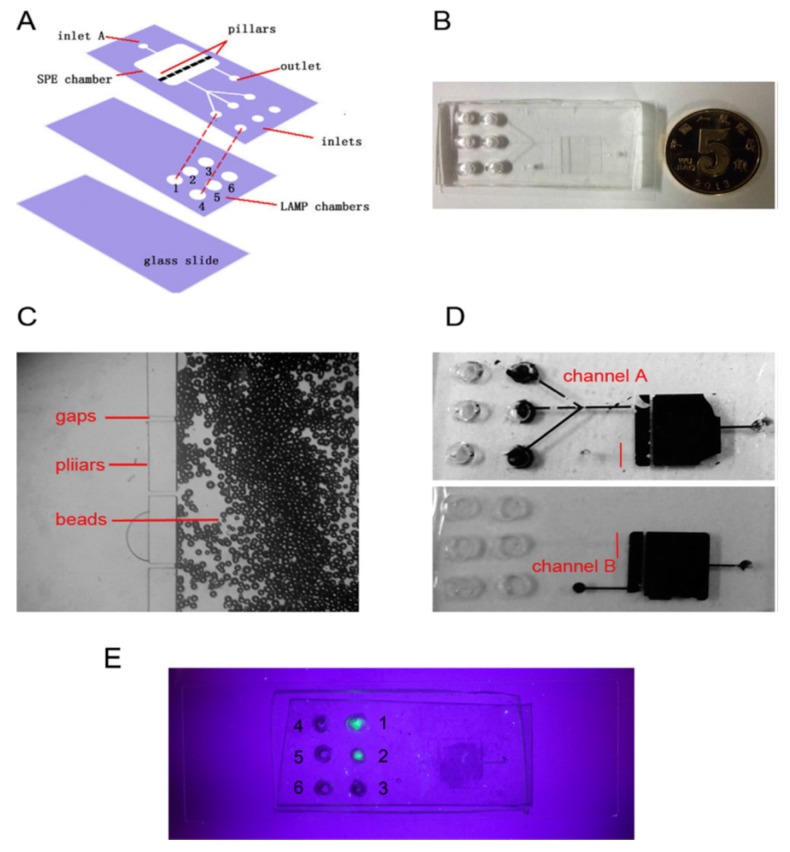
This microfluidic device consisted of two layers; (**A**) Schematic design of the device; (**B**) the real assembled device. The cells, DNA extraction reagents and silica beads are directed to the upper layer through the inlets to the SPE (Solid phase extraction) chamber. Following DNA extraction in the SPE chamber, the DNA samples are washed from the beads using distilled water to the second layer (LAMP chamber). The gaps on the micropillars are small enough to prevent bead transfer to the second layer; Subpanel (**C**) Shows the SPE chamber in which the beads stuck by the row of micro-pillars and the micro-gaps; (**D**): the solution flow in the chip is showed by an injection of black ink from inlet A flowed to channel **A** or **B**; the positive results can be seen by a, green fluorescence under the UV light (**E**) [123]. (This Figure is used from Elsevier with permission).

These concerns, however, are fairly improved by the invention of the LAMP method (Figure 15), in which the genome is primarily amplified under an isothermal condition (60–65 °C) within a period of 30–60 min [109]. Amplification of six different regions of the genome as a result of the application of 4–6 primers at the first screening step of LAMP makes this detection system more sensitive and specific than PCR [119,120]. Principally, the presence of a genome complementary sequence in each inner primer leads to the formation of stem-loop concatamers at the ends of each replicated fragment, which make a free 5′ end usable as a primer for further elongation on the strands. These repetitive reactions are finally led to the production of large quantity of stem-loop containing DNAs [121]. The genome amplification in LAMP is determined based on increase in the turbidity, as a result of reaction of pyrophosphates (released through the amplification) with magnesium (present in the tube), or change in the level of fluorescence illuminated from the fluorescent dyes (such as SYTO 9) intercalating with DNA [122]. In terms of automation of these devices, in addition to using fluorescence, release of H+ through amplification, which is directly proportional to the number of nucleotide used for each amplification, can be used as an ideal parameter as a proof of amplification and measurement of the numbers of amplification. In addition to the rapidity, low cost, high sensitivity and high specificity, LAMP is effectively applicable for the detection of microorganisms in the low quantity and quality samples, which is useful for direct detection of microorganisms even in the presence of clinical sample matrices [119,120]. Guo et al. designed an integrated double layer microfluidic chip (Figure 16) in which the DNA content isolated via silica beads were amplified by LAMP followed by a Calcein staining and UV light [123].

### 4.3. Genetic Polymorphism

Polymorphism in genetics denotes the presence of more than one alternate form (alleles) of inherited genetic contents of individuals in the population. Genetic polymorphism can be detected using different technologies, such as RFLP (Restriction Fragment Length Polymorphism) [124], T-RFLP (Terminal-Restriction Fragment Length Polymorphism) [125], AFLP (Amplified fragment length polymorphism) [126], RAPD (Random Amplified Polymorphic DNA) [127] and SSCP (Single-strand conformation polymorphism analysis) [128]. Restriction enzymes are a group of DNA endonucleases with the ability to cut the sugar-phosphate backbone of DNA at or near a very specific target sequence known as restriction sites, leading to produce several DNA fragments of individuals with a certain size [129]. Any mutation in a specific restriction site of a homologous DNA sequence resist the target against the corresponding restriction enzyme, which cause the production of fragments of different length [130]. RFLP is a combination of DNA digestion with one or more restriction enzyme(s), separation of fragments onto a gel through electrophoresis, and finally, detection of presence of specific fragments by a DNA probe [131,132]. 

AFLP is a more selective polymorphism technique in which, after digestion of a given DNA, the fragments are ligated to adaptors and then undergo pre-selective and selective PCR amplifications to reduce the fragment numbers [133]. The remaining fragments are run onto gel electrophoresis and the fragment profile is interpreted by software. Application of different types of adaptors enables this technique to study the polymorphism of hundreds of individuals in the same batch even without having any information about the microbial genomic sequence [133]. 

T-RFLP, however, works based on amplification of the gene target (mostly 16SrRNA) by fluorescent labelled primers, enzymatic digestion of the PCR products and, finally, the generation of the gel profile [134]. Due to the presence of fluorescent label at the 5^/^ end of fragments, a UV exposure will cause different peaks of various sizes and heights to appear, which is representative of the profile of a microbial community in a given sample [134]. T-RFLP, therefore, is a very useful and quick technique to investigate the microbial changes in an environment after treatment with a specific factor. However, it must be mentioned that this technique is not able to detect microorganisms in a sample, but only gives an idea of the levels of differences in whole microbial diversity of an environment [135]. 

**Figure 17:**
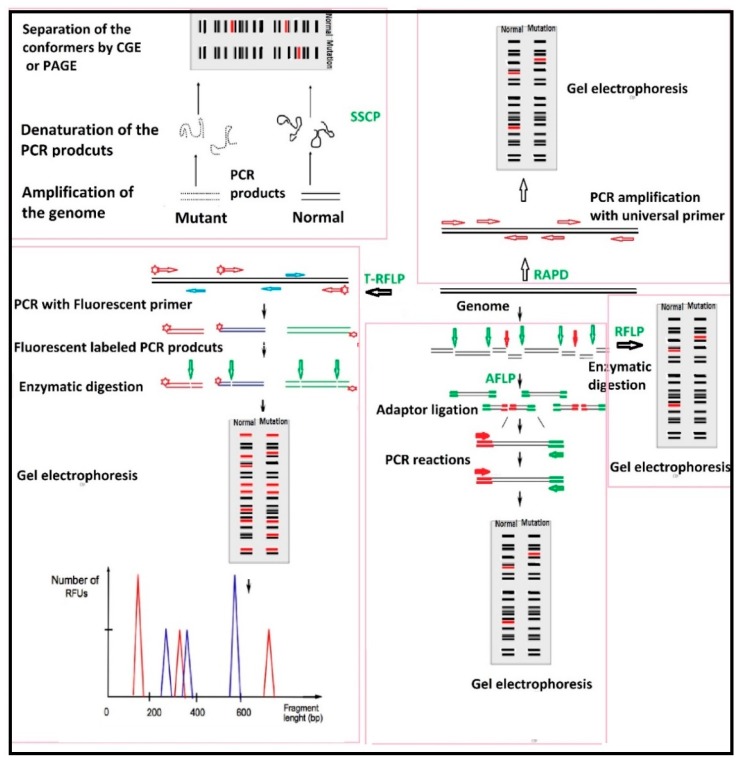
A schematic comparison of RFLP, AFLP, RAPD, T-RFLP and SSCP.

RAPD is a PCR-based polymorphism in which the given DNA is amplified with a universal primer (approximately 10 bp length), and the gel profile of individuals will be different as a result of mutations in the primer annealing sites (Figure 17) [136,137]. Because of using a universal primer, no specific knowledge of the microbial genome is required for the creation of microbial RAPD profile [136,137]. SSCP is an effective screening method to distinguish a specific genomic variation (like a specific strain) among a wide range of microorganisms. In this technique, following amplification of the genome, the PCR products are denaturised in the form of single strand DNAs. The presence of any mutation in the genome sequence leads to changes in the conformation of these single strand DNAs, which can be detected by separation onto polyacrylamide gel (PAGE) or Capillary Gel Electrophoresis (CGE) (Figure 17) [138].

While each polymorphism technique shows slight principal differences, the application of radioactive/fluorescent labels for detection of genome fragments enables automation of these technologies. However, these techniques are mostly used for detection of the presence of genomic changes in a strain in comparison to its normal sample, and to investigate the changes in the overall microbial profile of different samples. Therefore, these techniques are not recommended for the design of bio-chip devices intended for precise microbial detection in a sample.

### 4.4. Hybridization-Based Technologies 

Hybridization-based microbial detection technologies work based on the genome melting (de-hybridization) in high temperature and annealing (re-hybridization) of the complementary strands at lower temperatures [139]. Based on these techniques, a probe (a labelled short length single strand RNA/DNA complementary to a specific target sequence) binds specifically to its target on the genome through an annealing process, and the hybrid is detected by the (enzyme, radioactive or fluorescent) label on the probe [139].

**Figure 18:**
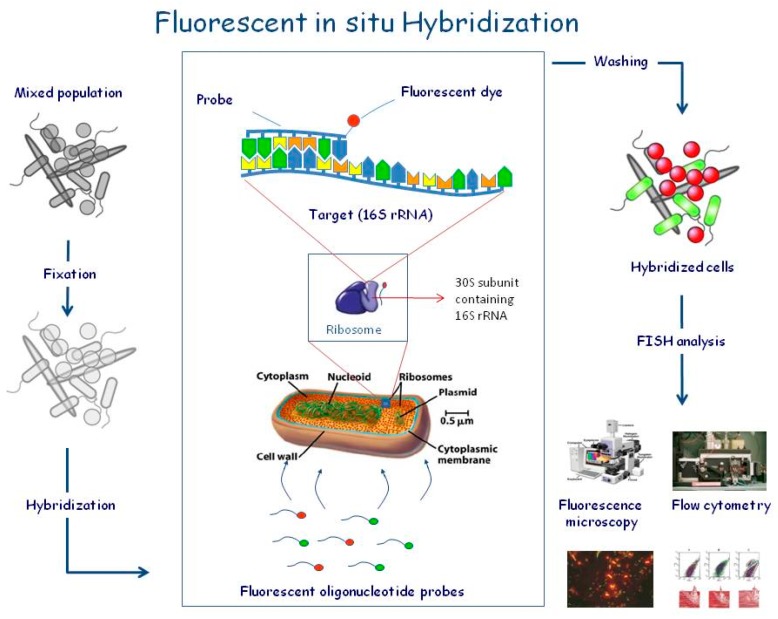
Fish (Florescent In Situ Hybridization).

FISH (Fluorescent In Situ Hybridization) (Figure 18) is a very sophisticated technique in which a fluorescent-labelled segment of RNA/DNA can be used to detect the presence of a specific complement of the genome in a sample and to locate the precise position of the complementary segment in the cell [140]. Application of several probes labeled to distinct fluorophore enables researchers to co-detect and co-locate multiple targets in the cells [140].

**Figure 19:**
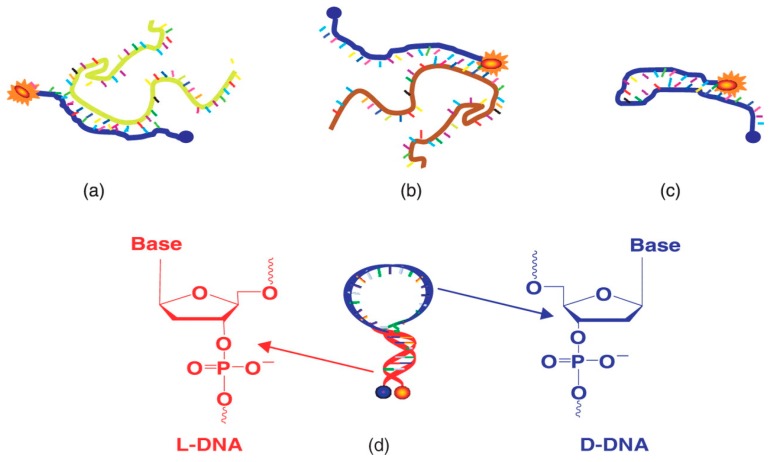
The structure and function of molecular beacons (MBs); MBs are target-specific single strand DNAs flanked with two complementary sequences labeled with a fluorophore (at one end) and a quencher (at the opposite end). (**a**) and (**b**) The MB-DNA target hybridization creates a distance between quencher and fluorophore, but (**c**) The hydrogen bonds between two complementary sequences at two ends make a hairpin structure in which any fluorescence released by the fluorophore is captured by quencher. (**d**) The production of d-DNA in the hairpin and l-DNA in the Double helix DNA.

A group of microbiological diagnostic biosensors is designed based on immobilization of a probe (a single strand DNA) complementary to the target DNA on the surface of a highly sensitive transducer [76]. The DNA hybridization with the target DNA can be detected by a fluorescence [141] or based on changes in a physical index such as electrical factors or acoustic waves [142]. Xi et al., designed a microfluidic device in which peptide nucleic acid (PNA) molecular beacons (MBs) were used as bacterial detectors based on DNA hybridization. A DNA MB is a single strand probe flanked with two complementary sequences to make a hair pin loop structure that, on one end, is labelled with a fluorophore and, on the second end, is attached to a quencher (Figure 19) [143]. As in real-time PCR, this structure causes the fluorescence produced by the fluorophore to be absorbed by the quencher. Hybridization with the target, however, increases the distances between the fluorophore and the quencher, and leads to illumination. Furthermore, based on alteration of the probe backbone with an electrically neutral compound (peptide nucleic acid; PNA) instead of a sugar phosphate backbone, they were able to improve its hybridization kinetics [143].

The DNA microarray is arguably the best fabricated and commercialized system for diagnoses based on DNA hybridization in which a group of each, out of millions, types of identical DNA probes are bound to specific spots of a solid surface, and their hybridization with a fluorescence-labeled DNA target is detected by a fluorescent scanner [144,145]. The integrity of hybridized DNA is detected by software base on the location of light emission.

While the majority of LoCs based on the DNA hybridization technique has been designed based on a light emission technology, the background light emission can affect their sensitivity and specificity. It is believed that the employment of electrical biosensors to detect the signals from DNA hybridization can improve the reliability of LoCs. While several types of electrical DNA microarrays have been designed, the majority of them have been limited to the research level due to the high noise levels exhibited by such devices [14]. Attachment of DNA probes onto the electrical circuits in a way to evoke a sensible electrical signal is a very challenging step in the design of DNA electric biosensors [14,76]. The chip bases can be covered with Streptavidin protein, which has the ability to bind the biotinylated DNA probe [15]. Using immobilization of DNA probes on a GMR (Giant MagnetoResistives), for instance, it is possible to design a CMOS biochip in which biotinylated DNA target/probe hybrid can bind to streptavidin-containing magnetic nanoparticles, and this complex can change the magnetic field disturbances [15,146]. Alternatively, mercapto-propyl-trimethoxsilane onto the surface of biosensors exhibit the ability to bind covalently to 5^/^ thiol-modified DNA probes [147]. Integration of CMOS with a successful electrical biosensor for detection of DNA hybridization is the gold key to design a successful LoC device with the ability of microbial diagnosis in a very complex sample.

### 4.5. Sequencing Dependent Techniques 

Gene sequencing, on the other hand, is a more accurate approach in which the sequences of usually a fragment of microbial genome (RNA or DNA) is determined, and the sequence is compared with the already present gene sequences in online universal databases, such as NCBI (National Centre for Biotechnology Information) [148]. Since the genetic elements of microorganisms are too variable, only a group of consensus genes, such as ribosomal DNA [149], gene *recA*, α and β submits of RNA polymerase (rpoA and rpoB) [150], Gyrase B subunit (*gyrB*) [151], which have been conserved or slightly changed throughout the evolution of microorganisms, are used to identify any phylogenetic relationships between microorganisms. The levels of similarities between two samples show the degree of relativeness between these samples. In addition to the accuracy, this technology enables researchers to identify and discover new uncultivable microorganisms in conventional cultures [151].

The existing commercial genome sequencers are classified into Sanger (an exemplar of so-called first generation sequencers), Illumina, Pyrosequencing, and Ion Torrernt (exemplars of second generation sequencers) plus Nanopore and Pacific Biosciences technologies (exemplars of third generation sequencers). The principal ability, advantages, and disadvantages of each sequencer have been described by Abbasian and colleagues [151].

Data collection in the second and third generation sequencing systems are significantly faster than the first generation techniques since these systems are designed based on DNA fragmentation, continuous cycles of enzymatic reactions, which are performed simultaneously on millions of fragments, and monitoring based on change in chemistry (such as pH and inorganic phosphor) or the emission of a special fluorescence [152]. A pre-amplification of the target genome by a specific primer increases the numbers of gene targets, and therefore, is the best way to reduce magnificently the time required for gene genome sequencing and data analysis, which are the main key reasons for limitation of the rate of microbial detection in these systems [153]. While all devices designed for next generation sequencing can be potentially used in LOC technologies, the small size of nanopore sequencers makes this technology perfect for the design of a fully automated miniaturized device for detection of microorganisms [154]. However, it is worth to remind that while the genome based methods show high sensitivity and specificity for microbial detection, their application are very questionable in terms of good laboratory practice (GLP) and biosafety assessments [155]. The limitations of these approaches for distinguishing the microorganisms with higher phylogenetic relationships and unreliability to the quality of gene sequences present in genome databases are mentioned as some of the shortages in the genome based studies. Furthermore, these methods are normally unable to distinguish viable microorganisms from the destroyed cells. However, treatment of the samples with a nuclease enzyme or an impermeable nucleic acid binding chemical to digest extracellular genomes or to prevent the DNA amplification may increase the possibility of detection of the genome obtained from viable cells.

## 5. Other Methods

In addition to genomic analysis, molecular based technologies for identification of microorganisms can be designed mainly based on the detection of genomic materials (RNA or DNA) and protein or fatty acid (FA) profiles of a cell and comparison of the results with information databases to analyze the similarity percentage [110]. The main advantages of these techniques are high sensitivity and very small sample requirements for detection of the microorganism of interest in a pure or mixed sample [110]. In addition to using only one or a few targets for detection of microorganisms, recently it has become possible to detect a single microorganism using whole gene materials (genomics), RNA profile (transcriptomics), protein profile (proteomics), FA profile or metabolic activity (metabolome) [156,157]. Furthermore, metagenomics is a technology to identify whole (known and unknown) microbial strains in a given environment based on the whole genetic materials of the sample [148]. The emergence of new technologies, such as advanced pattern amplification systems, next generation sequencing tools, genome/protein arrays, in-silico microbial metabolome, mass spectral protein and different automated chromatography systems have improved the sensitivity of microbial identification in a given sample [148,158,159].

### 5.1. Protein Based Methods

The protein based methods work either by immunological assays or gel electrophoresis. The immunological based techniques, such as ELISA (Enzyme Linked Immuno-Sorbant Assay), antigenic participation and agglutination assays and western blotting, which commonly are employed to identify microorganisms based on their surface epitopes, are very specific and all are limited to the presence of a specific antibody for the given protein as well as the sensitivity and specificity of the antigen-antibody reactions [160,161].

Since ELISA technology can be integrated with CMOS technology, we mainly focus on this subject. While routine ELISA kits used in diagnostic laboratories are able to detect only one specific microorganism or antigen in a given sample, CMOS technology offers the possibility of diagnosis of several microorganisms/antigens in only one kit [16]. In the other words, the application of CMOS-based biochips for immunological detection of pathogens improves the ability of identification of one or more microorganisms or their products within a complex matrix containing different microorganism [16]. Furthermore, in comparison to genome based assays that require genome extraction and gene replication, the immunological assays are short enough for direct detection of pathogens in urgent cases [162]. Therefore, the integration of antibodies with silicon dioxide passivated interdigitated electrodes is a very promising strategy to detect the presence of a specific An-Ab complex in a very quick (1–2 h) and sensitive device using changes in electrical indices [96]. In such devices, attachment of antigen on the antibody, which is coated on the surface of an interdigitated electrodes (IDE), led to changes the voltage and impedance values of the microelectrodes. While many attentions have been paid for the innovation of an integrative electrical device with the ability to detect Ab/Ag complex, its commercialization requires several technical upgrades and set up. Safavieh et al. [163], for instance, was able to design a HIV diagnostic paper microchip with a sensitivity of 107 virion per mL in which graphene-modified silver electrodes (GSEs) were conjugated with anti-HIV antibodies to sense the production of any Antigen (HIV)-Antibody complex in the clinical samples. Song et al., could also design a portable biochip system based on integration of a miniature CMOS sensor microarray and laser induced fluorescence (LIF) technology for single-bacteria detection in which the enzymatic product in ELISA test is excited by a laser beam, and the emitted fluorescent light is focused on a photodiode element of CMOS chip by passing through objective lens and optical mirror [60]. 

In addition, association of Gold Nanoparticle Probe (GNP) with antibodies and biotin enables these particles to bind at the same time to the specific microorganisms and Streptavidium-HRP (horseradish peroxidase), respectively, which generates visual light in the reaction with Tetramethyl Benzidine (TMB) [164,165]. Therefore, due to the generation of visual signals and the ability to read the result of microbial detection by naked eyes, this system does not require a specialist operator to interpret the results. To improve the sensitivity of this system, Ren et al. designed a magnetic LFIA magnetic system (mLFIA) in which the microbial cells are also captured with a magnetic nanoparticle, made of a Fe3O4/Au core equipped with antibodies against the specific bacteria. The creation of cell/Ironic nanoparticle facilitates the movement of these complex toward the GNP particles using a magnetic force [166]. While LFIA technology is a rapid, simple, labor free and cost effective approach for microbial screening in pathogenic and environmental samples, these devices are unable to detect a complex of antigens/microorganisms in a given sample that can be used as a multipurpose diagnostic device [166,167].

### 5.2. Bacteriocins

In addition to antibodies, it is possible to employ other molecules with specific affinity to microbial surface compounds. Bacteriocins are a group of compounds produced by specific bacterial strains to kill specifically other bacterial strains [168]. Therefore, the philosophy behind the application of these compound is to study the cell lysis of specific bacteria following treatment with specific bacteriocins. Nikkhou et al., for instance, employed two bacteriocins, Lysostaphin and colicin, for specific detection of *Staphylococcus aureus* and *E. coli*, respectively, onto a CMOS system. In this strategy, cell lysis caused by the bacteriocins was detected using potassium selective electrodes installed on CMOS systems to detect the increases in the potassium levels as a result of potassium efflux after cell lysis [169]. This system is applicable, however, for a narrow range of bacteria due to very specificity action of each bacteriocin on a range of bacteria [168]. Therefore, specific bacteriocins are required for detection of each bacterial species. Furthermore, since some bacteriocins can destroy a range of bacterial cells from different species (not only one strain), it is not possible to detect specifically the microbial strain. For instance, while Nikkhoo and et al. used colicin for detection of *E. coli*, a few bacteriocins exhibit inhibitory action on a range of bacteria; such as colicin against *Listeria monocytogenes*, *Salmonella* sp., [170] and subtilisin against some species of *Staphylococcus*, *Streptococcus, Enterococcus*, *Enterobacter aerogenes*, *Listeria monocytogenes*, *Pseudomonas, Porphyromonas*, *Kocuria, Escherichia*, *Shigella* [171]. Furthermore, the ability of bacteria to resist against bacteriocins [172,173,174] limits the application of bacteriocins for microbial detection.

## 6. Future Works

The design of hybrid CMOS/LOC devices for the detection of microorganisms in a given sample is a challenging subject for many researchers. These devices have the ability to detect microorganisms at the species, and even strain, levels. Operation speeds allowing 10–20 times shorter experimental periods and miniaturized profiles allowing for 100–1000 times less sample and reagents that are required for microbial detection are primary advantages of these technologies. All steps of an experiment, including sample collection, preparation, detection and data analysis are potentially targeted for design of a fully automated diagnosis. While many efforts have been devoted to develop this technology into a fast real-time means of microorganism mixture detection in (clinical, environmental, and industrial) samples, no significant developments have yet been achieved. The type, size, and nature of a microorganisms are all critical factors for the selection or design of a method for detection of that microorganism. Therefore, while the detection of microorganisms based on their morphological and biochemical features is too complicated and time-consuming, the engineers prefer to design genetic/immunogenic based biosensors. Since antibodies are specific to a special microbial strain, the antigenic-based microbial detection approaches are mainly limited to the presence of specific antibodies in the media. However, the application of universal 16SrRNA primers makes the genomic-based approaches very flexible, even for the detection of unknown microorganism. Therefore, since there is no idea about the type of microorganisms in complex samples, such as environmental samples, and in case of bioterrorism attacks, the genomic based approaches will be more useful for fast and effective microbial detection.

It must be noted that the commercialization of a device (see Table 1) depends mainly on the quality control and safety of the product, which, in turn, is determined by the test validation assessments to determine its precision, sensitivity, and robustness. While there is no limitation for the sensing options used in a detection method, the Fertilizer Safety Office suggests the application of an integrated approach in which both conventional and molecular techniques are used for to improve its sensitivity, specificity, and accuracy. Furthermore, since LoC devices usually need to be accompanied by a variety of ancillary machineries, such as pumps for microfluidic devices and amplifiers for electrodes, the bulkiness and final cost of these devices are the biggest challenges that bioengineers need to address to realize the technologies’ full potential. Integration of LoC devices with a universal pre-existing portable device, such as a smart phone, can be an applicable strategy to resolve these kinds of problems.

With the development of smartphones in our everyday activities and the feasibility of application of the smartphone apps by the users, many researchers and programmers have tried to bring these technologies into biology. However, the first commercial applications in this filed limited to the software that introduce the microorganisms, their effects in clinical/industrial/environmental microbiology and the best ways for treatment of the infectious disease [1]. Latest in this decade, engineers tried to integrate modern technologies to sense the presence of the whole cells or their components and metabolites by smartphones [2,3,4]. Park et al., for instance, reported a paper microfluidic device on which they loaded anti-*E. coli* antibody-conjugated beads. Any specific antigen (*E. coli*)-Antibody interaction on these papers led to immunoagglutination, which could be detected and quantified by analysis of Mie scattering from the digital pictures that were taken with a smartphone [2,5]. Later on, other immunochromatographic tests, such as fluorescence and luminescence, were joined with smartphones for the detection of enzymatic or antibody-antigen reactions [3]. Besides, Arts et al., were able to design a Luminescent Antibody Sensor (LUMABS) platform with the ability to detect antibodies directly in solution by a smartphone. LUMABS is made of a blue luciferase protein attached to a green fluorescent acceptor protein by a semi-flexible linker [6]. The presence of antibody in a solution disrupts the linker interaction between these two components, leading to the drops of bioluminescence resonance energy transfer (BRET) efficiency. They showed that this technique is able to detect antibodies at picomolar levels. As well, they showed that the manipulating of the structure of these LUMBAS enable specific detection of microorganisms in a solution. While all these designs depend on the employment of a specific/nonspecific factor for detection of microorganisms, Pei-Shih Liang et al., developed a smartphone-utilized biosensor with the ability to detect microorganisms on the surface of meat using Mie scatter, but without any requirement to antibodies or any other reagent [5]. They irradiated an 880 nm near infrared LED vertically to the surface of contaminated meat, and the signals scattered from the surface was collected by the digital camera of a smartphone and the gyro sensor at various angles. These types of technologies, however, defeat to distinguish microorganisms, and therefore, are only able to detect the presence of microbial cells on the subjects. While the integration of smartphone to the diagnostic systems is still in the first steps, it looks like this dream will come true very soon. For instance, Oxford Nanopore sequencer, which is able to sequence the genomic contents of microorganism, is a dedicated commercial approach with the ability to convert genomic codes into electronic data, which can be processed by smartphones [7].

## Figures and Tables

**Table 1:** Comparison between the commercialized Sensing Technologies.

Name of the Device/Kit	Designed by	Working Principle	Advantages	Limitations	Reference
Microbe sensor (BM-300C)	Sharp	Works based on a chemical reaction referred to Millard reaction in which reducing sugars and proteins are heated to produce Melanoidin with strong fluorescence in the presence of UV. This device heats the sample to proceed the Millard reaction inside the cells, and the presence of any Melanoidin is detected by UV.	- Fast detection period (10 min.) - Continuous measurement for ongoing monitoring of microbe counts - Compatible with Smartphone	Not usable for detection of types of microorganisms	(http://www.sharp-world.com/corporate/news/130926.html)
SYTO^®^ BC bacteria stain	Molecular Probes, Inc. (Eugene, OR, USA)	The quantity of bacteria can be detected by a SYTO^®^ BC stain, which is a high-affinity green fluorescent nucleic acid stain, and a Cytometer	Measure the numbers of both gram positive and gram negative bacteria.	- Not able to distinguish gram positive and gram negative bacteria - Not able to detect the type of microorganisms	https://www.thermofisher.com/order/catalog/product/B7277
Milliflex^®^ Rapid Microbiology Detection and Enumeration system	Millipore Sigma	Cells are trapped on a micro-filter and the number of cells are measured by Bioluminescence-ATP (adenosine triphosphate)	- Automated device for quantification of whole microbial cells	- Requires a long incubation time to grow microorganisms - Not able to differentiate microorganisms	http://www.emdmillipore.com/CA/en/product/Milliflex-Rapid-Microbiology-Detection-and-Enumeration-system,MM_NF-C10711
Celsis^®^ systems	Charles River	The proprietary adenosine triphosphate (ATP) bioluminescence technology	- Automated device for quantification of whole microbial cells	- Requires a long incubation time to grow microorganisms - Not able to differentiate microorganisms	https://www.criver.com/products-services/qc-microbial-microbial-detection/microbial-detection-instruments?region=3601
Bactometer Microbial Analyzer	Biomerieux	These systems use Colorimetry od grown bacteria in defined media	- High discrimination between species - Antibiotic susceptibility testing	- Not real time - Not able to detect unknown microorganisms	http://www.biomerieux-usa.com/clinical/vitek-2-healthcare
PCR (Polymerase Chain Reaction)	Several companies (such as Eppendorf, Bio-Rad and Ampicon)	Detection of microorganisms based on amplification of target gene	Highly accurate	- Not real time - Not able to distinguish dead and alive cells - Not for qualification	[151]
Real-time PCR	Several companies (such as Eppendorf, Bio-Rad and Ampicon)	Detection of microorganisms based on amplification of target gene	- Highly accurate - Quantitative	- Not real time - Not able to distinguish dead and alive cells - Not able to separate different microorganism unless in combination with other techniques	[151]
Next generation Gene Sequencing systems	Several companies-454 Life Science, Ion Torrent-Illumina-Oxford NanoporeSOLiD,	Detection of microorganisms based on amplification of target gene	- Highly accurate - Quantitative - Able to detect the presence of different microorganisms in one sample	- Expensive - Not real time - Required bioinformatic analysis	[151]
